# Goal setting in people with low back pain attending an education and exercise program (GLA:D Back) and the impact of demographic factors

**DOI:** 10.1186/s12891-024-07450-w

**Published:** 2024-04-27

**Authors:** Mette H.M. Gregersen, Kristine R. Nielsen, Nana H. Lynge, Bibi D. Heiberg, Jan Hartvigsen, Greg Kawchuk, Alice Kongsted

**Affiliations:** 1 Center for Muscle and Joint Health, Department of Sports Science and Clinical Biomechanics, University of Southern Denmark, Campusvej 55, Odense M, 5230 Denmark; 2 Chiropractic Knowledge Hub, Campusvej 55, Odense, Denmark; 3 Department of Physical Therapy, Faculty of Rehabilitation Medicine, University of Alberta, Corbett Hall, 8205 114 St NW, Edmonton, AB Canada

**Keywords:** Low back pain, Self-management, Patient led goal setting, International Classification of Functioning, Disability and Health (ICF), Demographic factors

## Abstract

**Background:**

Individual goal setting is a fundamental element in self-management supportive interventions, serving to guide actions and enhance motivation for engagement. Despite this, little is known about the goals people with back pain have and to what extent these differ across genders, age groups and geographical location. This study aimed to elucidate this by first describing individual goals set by Danish and Canadian participants in a self-management intervention for people with back pain using the ICF framework; then, determining what proportion of these goals met criteria for being specific, measurable, acceptable, and time bound, and finally, by investigating differences between countries, sexes, and age groups.

**Methods:**

In a cross-sectional study conducted August 2018 to June 2020, 394 Danish and 133 Canadian (Alberta Province) participants defined their individual goals of participating in a self-management programme involving patient education and supervised exercises. The goals were linked to the ICF framework. Distribution of goals was compared between countries, sexes, and age groups.

**Results:**

Goals most often related to the ICF component of ‘Activity and Participation’. The most prevalent goals were “Walking” (DK: 20%; CA: 15%) and “Maintaining a body position” (DK: 17%; CA: 22%). Only few goals differed between populations, age and sex. All elements of SMART goal setting were recorded for 88% of Danish and 94% of Alberta participants.

**Conclusions:**

People with low back pain attending a self-management programme established goals according to the SMART criteria and focused primarily on activity. Goals were similar across countries and showed few differences across sex and age groups. The high number of different goals points to the need for individualised person-centred care.

## Introduction

Low back pain (LBP) affects more than 600 million people globally [1]. Most episodes of LBP are short-lasting with few consequences, however, in some, it develops into a chronic disabling condition characterised by complex interactions between biological factors, pain, behaviours, cognitions, emotions, and the social context [2]. To reduce disability from chronic LBP, people are helped by having the ability to manage all the aspects of living with a chronic condition, and an important part of their health care is self-management support [3, 4].

Self-management support involves providing patients with knowledge, skills, and tools to live well with a chronic condition [5, 6]. This includes the use of behaviour change techniques (BCTs) such as goal setting, action planning, problem-solving, and graded tasks [7–9]. Goal setting is a tool for identifying valued activities that can guide care and support patient motivation [10–12], and has shown promise for improving outcomes in people seeking care for chronic LBP and osteoarthritis [13–15].

People seeking care for musculoskeletal conditions report various goals and the International Classification of Functioning, Disability and Health (ICF) is feasible for classifying these patient-defined goals in healthcare [16–19]. The ICF is the WHO framework for describing health and disability at both individual and population level. It provides a common language that allows for systematic classification of patient goals beyond medical aspects and comparing them across conditions, settings, interventions, and patient subgroups.

Setting specific, measurable, achievable, relevant, and time-bound (SMART) goals is a well-described method for systematic goal setting [20] with a focus on what you specifically want to change [21]. Goal setting using a SMART approach is used as part of GLA:D Back, a structured programme delivered in physiotherapy and chiropractic practises in Denmark and the province of Alberta in Canada [22–24]. In GLA:D Back the SMART acronym is interpreted as agreeing on a Specific goal, a way to Measure it, the Acceptable level of discomfort when achieving the goal, the Relevance of the goal, and a Time frame to reach the goal [22]. GLA:D Back consists of individual consultations for goalsetting and clinical testing, two 1-h group-based patient education and 16 exercise sessions designed to support self-management in people with chronic LBP. Clinicians are trained in delivering GLA:D Back in a 2-day course and provided with materials to facilitate the delivery [25].

Existing evidence suggests that patient goals for LBP care differ across age groups and genders [19, 26]. However, studies are not directly comparable due to differences in patient populations, methods for goal setting, interventions and societal/cultural settings making it unknown to what extent differences observed across studies are due to any of these factors. By comparing goal setting between two countries where the same target population undergo the same intervention it is possible to assess if cultural differences make a difference to the type of goal and goal setting process.

Understanding the goals people with LBP have and how they may differ across countries or patient subgroups is important for informing the development of patient-centred care [27]. The objectives of this study were to: (1) Describe the goals set by Danish and Canadian patients enrolled in the GLA:D Back program by linking goals to the ICF framework, (2) determine what proportion of the goals defined in GLA:D Back met the SMART criteria, (3) investigate if the type of goals described differed between Danish and Canadian patients, and (4) investigate if the type of goal was associated with age and sex.

## Methods

### Overview

This was a cross-sectional observational study based on data from the Danish and Canadian GLA:D Back registries collected between the 6th of August 2018 and the 9th of June 2020 [23, 24]. In dialogue with a clinician, patients defined their personal goals according to an adapted SMART model when they enrolled in the program. We linked these goals to the ICF and the distribution on ICF classes were compared between countries and patient groups. The study is reported according to the STROBE statement [28].

### Setting

At the time of data extraction, the GLA:D Back program had been implemented in 194 physiotherapy and chiropractic clinics in Denmark [29] and had been tested for feasibility in 19 clinics in Alberta, Canada [30]. SMART goal setting was taught to the clinicians in a combination of lecturing and small group discussions in a 30-minutes session within the GLA:D Back training course [25]. Clinicians were introduced to the SMART approach [21] and trained to encourage patients to define their goals towards a function rather than a pain- or structurally related goal [23].

### The goal setting process

Goal setting was part of the first individual session of the GLA:D Back intervention, where the clinician prompted patients to define a goal related to activity or participation which was registered as four elements of a SMART goal [22]. A “Specific” goal is tangible leaving no doubt about what needs to be accomplished [21]. In this project, all goals were automatically classified as “specific” and patients with no goals were excluded from the analysis. “Measurable” means that goal achievement can be quantified and progress can be monitored [21]. In GLA:D Back the “A” in SMART was modified from “Achievable” to “Acceptance of discomfort” to facilitate a dialogue about dealing with potential discomfort and pain provocation achieving a goal [21]. The “R” in SMART representing “Relevance” was not registered in the database in GLA:D Back as it was assumed from the developers of GLA:D Back that the discussion of the patients’ goal between clinician and patient would lead to a relevant goal [22]. Therefore, the “R” is not further described or analyzed. “T” is used for “Time bound” to define when to monitor progress [21].

### Participants

GLA:D Back was designed for people with chronic or recurrent non-specific LBP in need of improved self-management. Participants were people seeking care from clinicians who had participated in the GLA:D Back training. Other than age ≥ 18 years, no firm inclusion criteria were defined and inclusion was decided in a dialogue between patients and clinicians [23]. The clinics were mainly private practices with patient self-payment, which prevented some eligible patients from participating [31].

**Fig. 1:**
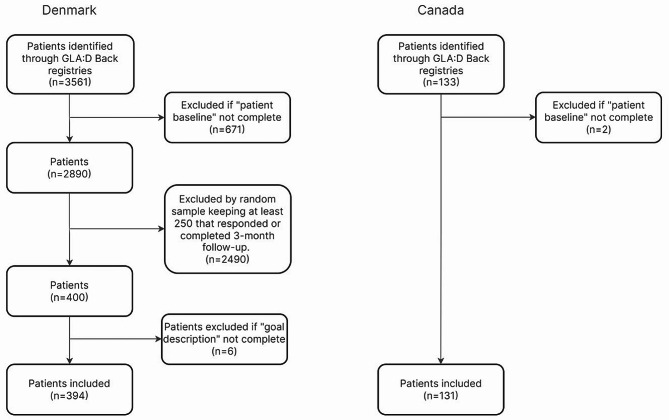
Patient flow chart

Data from 3561 patients in the Danish GLA:D Back registry was available, with 2890 (81%) completing baseline questionnaires. A random sample of 400 patients was created from the 2890 records using a random sample in the statistical software STATA. Six records had no goal registered and were excluded leaving a Danish sample of 394 patients (Fig. 1). The Canadian GLA:D Back registry covered 133 patients at the time of data extraction, whereof two had no goal registered and were excluded leaving a Canadian sample of 131 patients available for analysis (Fig. 1).

A total sample of 500 participants was realistic to link to ICF within the timeframe of the study and would provide 10 observations even in goal categories chosen by only 2% of the sample.

### Data collection

When enrolled in the GLA:D Back program, the clinician registered the patient into the electronic data registry in REDCap where clinicians also reported the goals according to the SMART criteria, (with relevance “R” being excluded) [22] (Table 1).

**Table 1:** Definition of the used SMART variables including measure and example for each variable

SMART Variables	Description	Example
Specific goal description	Describe activity (free text)	Stand at work desk without sleeping sensation in legs
Measurement	How much, time or distance and/or how often, for example, times per week? (free text)	20 min. 5 times during an 8-hour shift
Acceptance of discomfort	Degree of discomfort associated with achieving goals (0–10; 0 = None, 10 = Worst imaginable)	8
Time frame	How many weeks are set to reach the goal? (weeks)	8 weeks

A baseline survey was then emailed to the patient. It collected demographic information, information on LBP history and other clinical characteristics (not all part of this study) [23] (Table 2).

**Table 2:** Patient reported variables collected at baseline survey via email [23, 45]

Variables	Description	Scale
Sex	Extracted from personal identification number	Female | Male
Age	Extracted from personal identification number	18–39 years | 40–59 years | 60 + years [46]
Pain duration	How long has it been since the current pain began?	0–2 weeks | 2–4 weeks | 4–12 weeks | 3–12 months | More than 1 year
Previous episodes of LBP	Before this episode of LBP, how many episodes of LBP have you been treated for in the last 2 years?	No | 1 episode | 2–3 episodes | More than 3 episodes
Back pain	Pain intensity within the last week in Numeric Rating Scale (NRS).	0 = No pain to 10 = Worst imaginable
Disability	Current activity limitation on Oswestry Disability Index (ODI).	0–100; higher scores reflect more disability

### Data analysis

#### Linking of GLA:D Back SMART goals to ICF

**Fig. 2:**
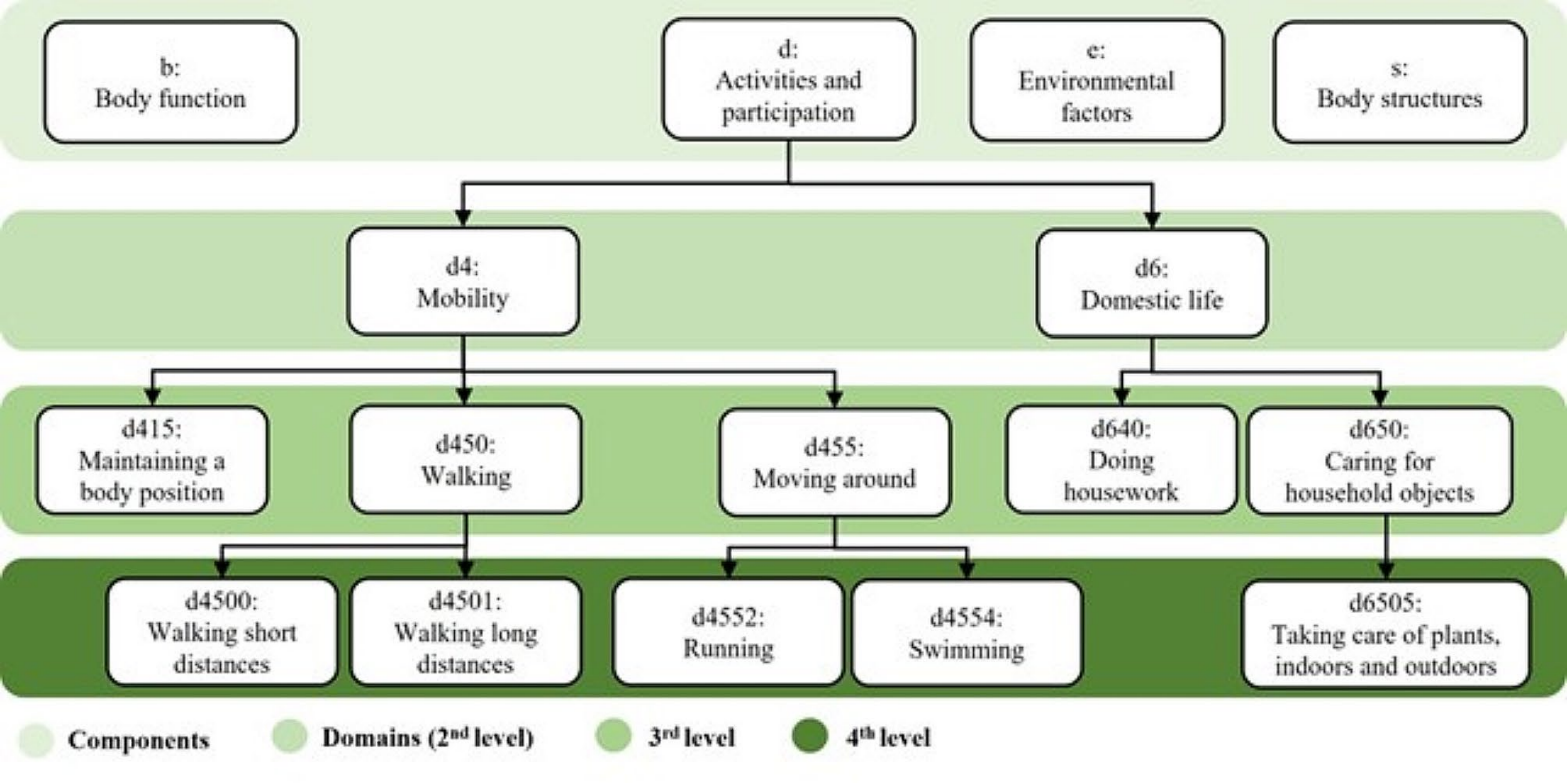
Example of division from components of the ICF to Four-level Classification

The ICF is a hierarchically organised classification system containing more than 1450 categories covering all aspects of disability and functioning. All categories are sorted in to components of body functions (*b*), structures *(s*), activities and participation (*d*), and contextual factors including environmental factors (*e*) and personal factors. These components are further divided by unique alphanumeric codes organized into domains (2^nd^ level) and 3^rd^ and 4^th^ level categories [18] (Fig. 2). Only exception is the personal factors that are not yet classified into ICF categories due to lack of clarity of personal factors including societal and cultural diversity [32].

The most detailed level of classification used in the analyses was the 3^rd^ level classification (one letter and three digits) to avoid very small groups of categories.

The ICF allows for designating some domains to both Activity and Participation using one of four principles [18]. In this project the same component (d) were used for both Activity and Participation.

Each goal was linked to the ICF following the ICF Linking Rules part of which is that you should identify the main concept(s) and additional concepts and link them to the most precise ICF category [33] (Table 3).

**Table 3:** Definition of ICF components with examples [18]

ICF Component	Examples from GLA:D Back (original text shortened)	Main concept	Lowest ICF category
Body Function and Structure (B & S). Def.: *The physiological functions of body systems with body structures referring to the anatomical parts of the body.*	Sleep well.	To sleep with good quality	b1343 Quality of sleep Def.: *Mental functions that produce the natural sleep leading to optimal physical and mental rest and relaxation.*
Activity and Participation (A & D). Def.: *The complete range of domains denoting aspects of functioning from both an individual and a societal perspective.*	Get out of a car. Engage in a football match.	Getting out of sitting position from car Engage in organized game.	d4103 Sitting Def.:*Getting into and out of a seated position and changing body position from sitting down to any other position, such as standing up or lying down.* d9201 Sports Def.: *Engaging in competitive and informal or formally organized games or athletic events, performed alone or in a group, such as bowling, gymnastics or soccer.*
Contextual factors: Environmental factors and Personal factors (E & P). Def.: *Making up the physical, social, and attitudinal environment in which people live and conduct their lives.*	Avoid increasing consumption of medicine	Intake of products or substances for personal consumption	e1101 Drugs Def.: *Any natural or human-made object or substance gathered, processed or manufactured for medicinal purposes, such as allopathic and naturopathic medication.*

The linking was performed manually using the software package NVivo [34] as a tool to organize the linking of the goals and make it easy to compare the linking between authors.

For each GLA:D Back goal, the purpose of information was identified as a meaningful concept from the goal descriptions, which supported linking to the most precise ICF category [33]. If more than one meaningful concept was captured, for example if a patient described both an activity goal and a goal of pain reduction, each concept was linked to the ICF separately.

All Danish goals were first linked to an ICF component by two of the authors (MHMG and BDH) separately. Then, the linking was systematically reviewed by the first author and disagreements discussed between the linkers. If consensus could not be reached, a decision was made based on a third researcher’s assessment. Finally, goals were designated to a domain and linked to a two or third-level item by KRN and NHL. The linking of Canadian goals to an ICF component were assessed only by the first author based on a high level of agreement obtained when linking the Danish data to an ICF component (agreed about 388 goals/394 goals = 98.5%). The designation to a domain and linking to a two or third-level item of the ICF for the Canadian goals were performed by MHMG, KRN and NHL. All authors involved in the classification of goals had completed the ICF e-learning Tool [35].

### Defining goals as adherent to the SMART Approach

A goal was classified as SMART adherent if the clinician had completed all four registry elements (specific goal, measurement, acceptance of discomfort, and time frame). The goals set by the Danish patients were classified according to SMART separately by two authors (MHMG and BDH) and systematically reviewed by the first author. There were no disagreements between the researchers. The classification of data from Canadian patients were therefore assessed only by MHMG.

### Statistical analysis

Differences in the distribution of goals between Denmark and Alberta were tested with a chi^2^-test. When comparing ICF-domains between countries, we ignored categories with less than 10 observations. A primary goal was not defined in the registry if participants had more than one goal and all goals were considered equal in the analyses.

Differences in SMART adherence between countries was investigated by comparing the proportion of patients for whom all four elements were registered using a chi-squared test.

Associations between the type of goal, age and sex were estimated using logistic regressions with the goal registered for the patient (yes/no) as dependent variable and sex or age as the independent. Analyses of sex were adjusted for age and vice versa and both for population. When associations were in opposite directions for Denmark and Alberta, we also included an interaction term between population and the dependent variable.

## Results

### Study sample

Most patients in the Danish cohort were females, with a mean age of 57.2 years, and more than 50% reported back pain that had lasted more than one year (Table 4). The Canadian participants were slightly younger and reported less disability than the Danish.

**Table 4:** Descriptive patient characteristics

Baseline characteristic	Denmark (*n* = 394)	Alberta (*n* = 131)
Sex, n (% female)	266 (69%)	73 (65%)
Missing values, n	9	19
Age, mean (std. deviation)	58.3 (13.0)	55.9 (14.0)
Missing values, n	7	19
Pain duration, n (%)		
0–4 weeks	30 (8.0%)	17 (15.3%)
4–12 weeks	41 (10.9%)	9 (8.1%)
3–12 months	87 (23.1%)	18 (16.2%)
> 1 year	218 (58.0%)	67 (60.4%)
Missing values, n	18	20
Previous episodes, n (%)		
0–1	68 (17.8%)	24 (21.4%)
2–3	148 (39.0%)	27 (24.11%)
>3	164 (43.2%)	61 (54.5%)
Missing values, n	14	19
Backpain baseline (NRS), median (25th − 75th)	5 (4–7)	5 (3–7)
Missing values, n	16	19
ODI sum baseline, median (25th − 75th)	24 (16–34)	13 (9–17)
Missing values, n	27	48

NRS: Numeric Rating Scale. ODI: Oswestry Disability Index

### ICF categorised goals

**Fig. 3:**
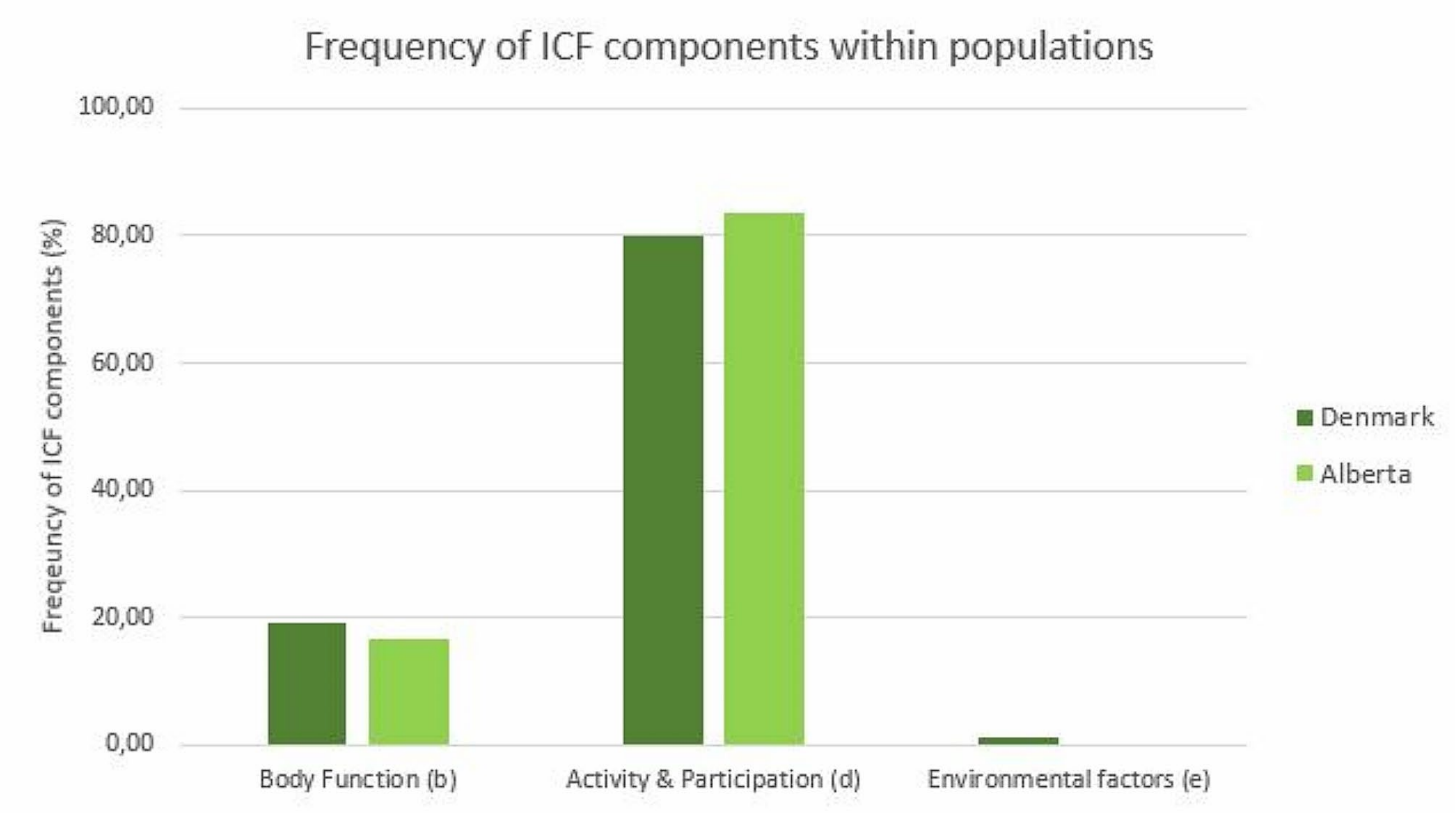
Comparison of frequencies of ICF Components between Denmark and Alberta

More than one goal was registered in 87/394 (22%) of Danish participants and in 11/131 (8%) Canadians resulting in a total of 638 individual goals (DK:493; CA:145). The participants’ goals most often related to the ICF component ‘Activity and Participation’ (DK: 80%; CA: 83%), followed by ‘Body Function’ (DK: 19%; CA:17%) and 5 goals (1%) from ‘Environment’ in the Danish sample (Fig. 3).

**Fig. 4:**
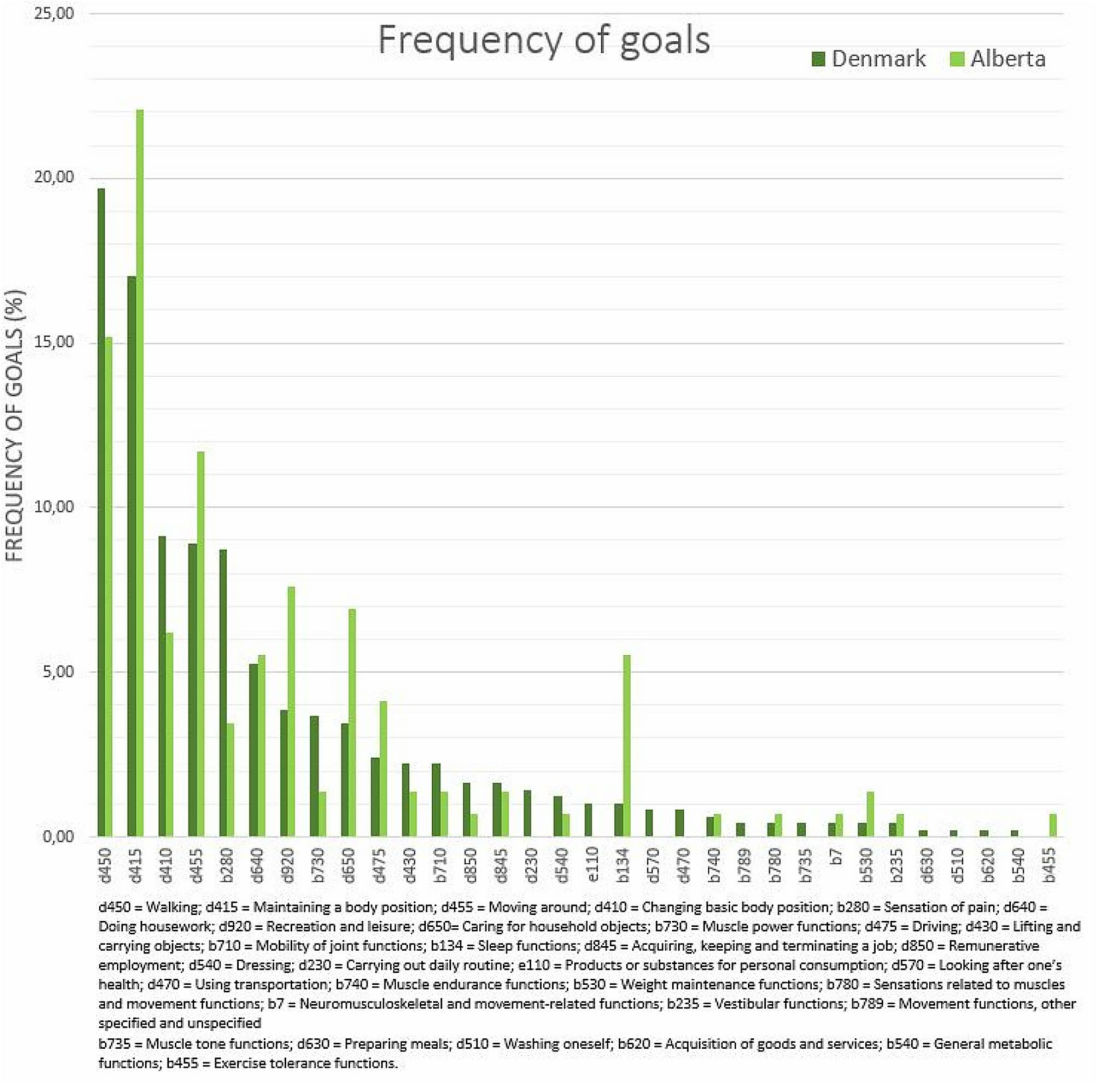
Frequencies of the goals set by GLA:D Back participants

The most prevalent goals at 3^rd^ level classification were “Walking” (DK: 97 (20%); CA: 22 (15%)), “Maintaining a body position” (DK: 84 (17%); CA: 32 (22%)), “Moving around” (DK: 44 (9%); CA: 17 (12%)), and “Changing basic body position” (DK: 45 (9%); CA: 9 (6%)) (Fig. 4). In Demark, “Sensation of pain” constituted 43 (9%) of the goals, while in Alberta this was 5 (3%). “Recreation and leisure” represented 11 (8%) of goals in Alberta and only 19 (4%) in Denmark. The most frequent goals from the Body Function component were “Sensation of pain” in Denmark (43 (9%)) and “Sleep functions” in Alberta (8 (6%)).

### SMART criteria

**Fig. 5:**
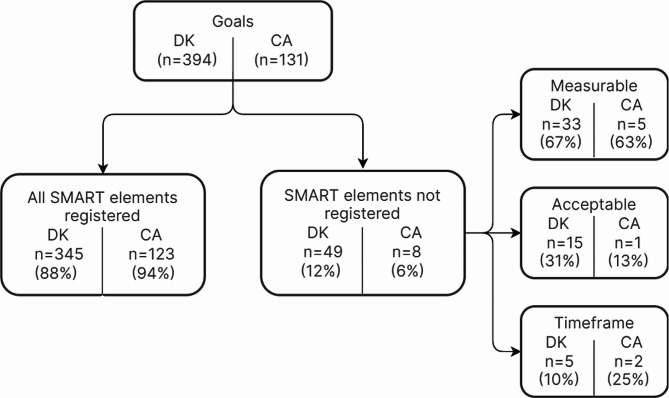
Flowchart of registration of SMART variables by clinician. Lack of registration furthest to right

All four SMART elements were registered for 345 (88%) of the Danish and 123 (94%) of the Alberta patients (Fig. 5). In both countries, the most frequently missing element was “measurable” (DK: 33 (67%) and CA: 5 (63%)), i.e. deciding how goal achievement would be measured. Three Danish patients (0.8%) lacked registration in more than one component, and none of the Canadian patients lacked registration of more than one element.

### Association of type of goal with sex and age

**Fig. 6:**
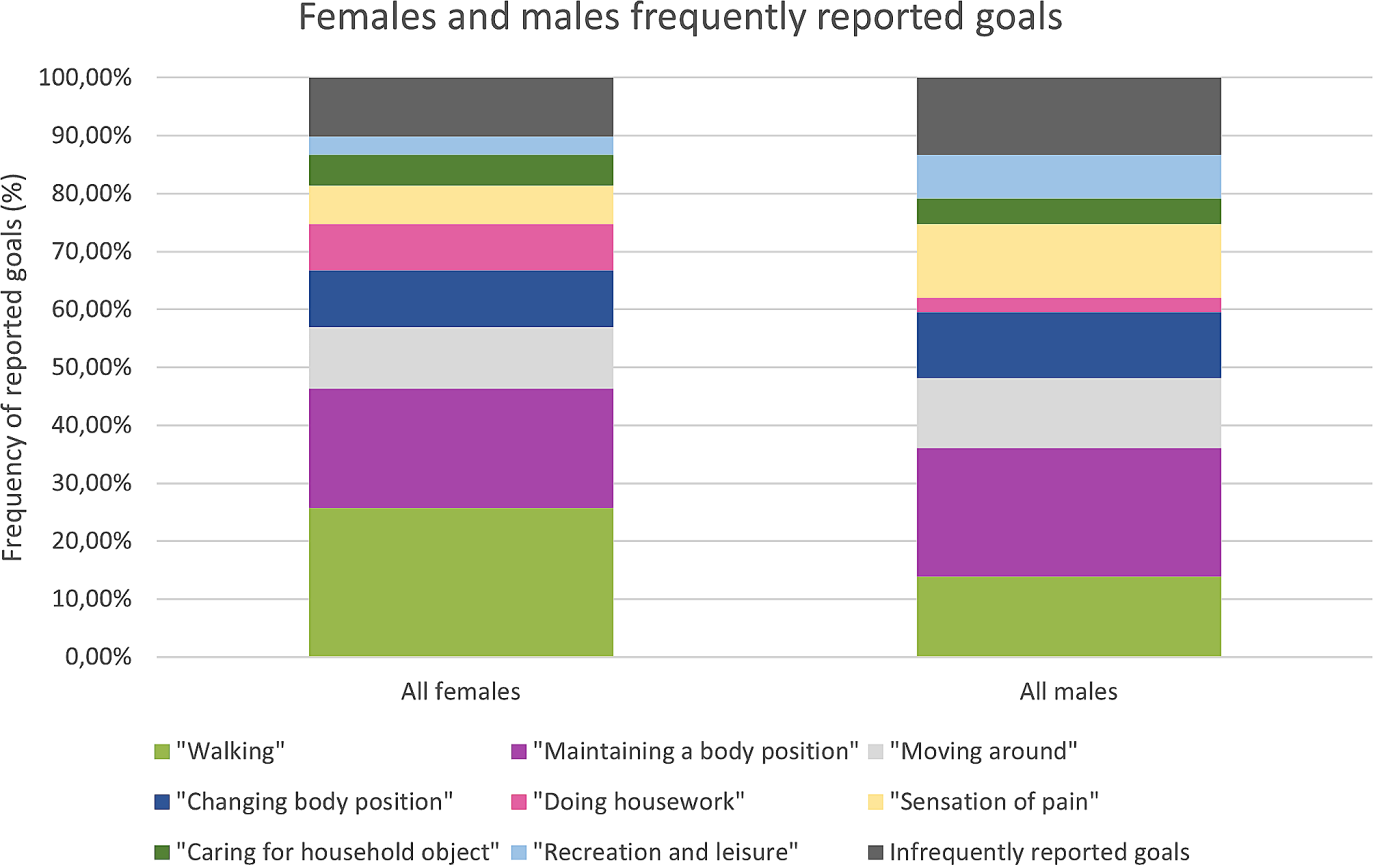
Comparison of frequently reported goals between females and males

Females more often had goals related to “Walking” and “Doing housework”, whereas males more frequently had goals related to “Recreation and leisure” and “Sensation of pain” (Fig. 6). For other frequently reported goals we did not observe any systematic sex differences.

Finally, regarding age groups “Walking” was a more frequent goal, and “Maintaining a body position” was a less frequent goal with increasing age (Table 5). Also “Recreation and leisure” was observed less frequently in the oldest group when compared to the other age groups. For other goals, no substantial differences were observed across age groups.

**Table 5:** Association of type of goal with age (adjusted for sex and population)

	Denmark (95% CI)			Alberta (95% CI)			Total sample (95% CI)		
Goal description	**< 40** *N* = 30	**40–60** *N* = 159	**> 60** *N* = 198	**< 40** *N* = 15	**40–60** *N* = 45	**> 60** *N* = 52	**< 40** *N* = 45	**40–60** *N* = 204	**> 60** *N* = 250
Walking	10% (3.3–22.3)	15.7% (10.8–22.3)	32.3% (26.2–39.2)	0% (-)	8.9% (3.3–21.6)	25.0% (15.0–38.6)	6.7% (2.2–18.8)	14.2% (10.0–19.7)	30.8% (25.4–36.8)
Maintaining a body position	46.7% (32.8–37.2)	20.1% (14.6–27.1)	15.7% (11.2–21.4)	40% (19.0–65.5)	26.7% (15.7–41.5)	21.2% (12.0–34.5)	44.4% (32.7–61.2)	21.6% (16.5–27.8)	16.8% (12.7–22.0)
Moving around	16.7% (7.1–34.4)	14.5% (9.8–20.9)	8.1% (5.0–12.8)	6.7% (0.9–35.7)	11.1% (4.7–24.2)	10.7% (5.2–23.6)	13.3% (6.1–26.7)	13.7% (9.6–19.2)	8.8% (5.9–13.0)
Changing basic body position	3.3% (0.5–20.3)	10.7% (6.7–16.6)	12.1% (8.3–17.5)	13.3% (3.3–40.9)	8.9% (3.3–21.6)	5.8% (1.9–16.6)	6.7% (2.2–18.8)	10.3% (6.8–15.3)	10.8 (7.5–15.3)
Doing housework	3.3% (0.5–20.3)	6.3% (3.4–11.3)	7.1% (4.2–11.6)	6.7% (0.9–35.7)	4.4% (1.1–16.3)	5.8% (1.9–16.6)	4.4% (1.1–16.2)	5.9% (3.4–10.1)	6.8% (4.3–10.7)
Recreation and leisure	6.7% (1.7–23.2)	4.4% (2.1–9.0)	3.4% (0.8–5.3)	6.7% (0.9–35.7)	15.6% (7.5–29.4)	3.9% (1.0–14.3)	6.7% (2.2–18.8)	6.9% (4.1–11.3)	2.4% (1.1–5.3)
Caring for household object	0% (-)	3.8% (1.7–8.2)	5.1% (2.7–9.2)	13.3% (3.3–40.9)	4.4% (1.1–16.3)	9.6% (4.0–21.3)	4.4% (1.1–16.2)	3.9% (2.0–7.7)	6% (3.6–9.7)
Sensation of pain	13.3% (5.1–30.7)	9.4% (5.8–15.1)	10.1% (6.6–15.2)	6.7% (0.9–35.7)	2.2% (0.3–14.4)	3.9% (1.0–14.3)	11.1% (4.7–24.1)	7.8% (4.9–12.4)	8.8% (5.9–13.0)

## Discussion and Conclusion

### Discussion

Most frequently goals for people participating in a self-management program for chronic LBP in Denmark and Alberta were related to the ICF activity and participation components and included the domains “Walking”, “Maintaining a body position”, and “Moving around”. The goals were very similar between people in Denmark and Alberta, Canada indicating that the goal setting process was conducted similarly in both Denmark and Alberta and people with LBP have similar goals despite any cultural differences present between these locations. The similarity continues across sex and age groups. Noticeably, however, women more often had goals related to walking and household, whereas related to leisure time activities and pain were more common among men. Clinicians adopted the concept of goal setting after a short introduction and made registrations according to SMART for almost all participants.

Goals described by participants in the GLA:D Back program were mainly about ‘Activity/Participation’ with few goals to improve ‘Body Function/Structure’ similarly to findings in the study by Lohmann et al. [36], a German study using the ICF to identify the rehabilitation goals of patients in early post-acute rehabilitation This is unlike primary care studies from Norway and the Netherlands where goals classified as ‘Symptom’ or ‘Body Function/Structure’ were most common amongst patients with back pain [19, 37]. This difference may relate to goal setting being used to inform delivery of the intervention in GLA:D Back [10], whereas in the other studies goals were measured for research purposes [19, 37]. In GLA:D Back, clinicians were taught that pain and structure goals may not be useful as drivers of engagement to achieve better pain self-management, and they were asked to explore with patients “What would change in your life if your pain was reduced?” [23]. Thus, participants in GLA:D Back may initially have stated goals related to structure and pain reduction but changed those to activity goals when this was facilitated by the clinician. This aligns with findings from an Australian study that trained a physiotherapist in facilitating goal setting using SMART, which found physical activity goals to be the most common category of goals [38].

We investigated goal setting as part of a structured intervention in routine primary care practices and included a large sample from the Danish dataset while having a minor sample of data from Alberta due to the GLA:D Back only being enrolled in the province of Alberta at the time of data extraction. In total a large sample combining the two populations was analysed. The goals where first linked to the overall ICF component, and then to the lowest possible ICF classification used for analyses to avoid very small groups. This provides insight into what exact activities patients with LBP perceive to be restricted in.

The main limitation of the study was that fidelity to the goal setting process was not investigated as they took place during daily clinical practice across many clinics. The SMART approach was feasible for clinicians to use for goal setting, but insufficient clinician guidance when using SMART goal setting can be a limitation as it is subjective what defines a goal as ‘specific’ and the goals registered lacked detailed information. Therefore, ‘Activity/Participation’ goals were defined as intended, but perhaps specific goals such as “Walking” and “Changing or sustaining a position” in reality are just indicators of a value-based goal, for instance wanting to walk or sustain a position to participate in social activities or work [13]. Recognizing that LBP can substantially affect all aspects of life, we would suspect that a further dialogue about these goals may have revealed more goals reflecting emotional and social consequences of pain.

Due to lack of more detailed information, the analyses were limited to the 3^rd^ level domain of ICF. It might be preferable to link to a lower level of ICF, but we doubt this would provide more relevant information. The goal setting process would have been challenging for some patients unprepared to actively engage in defining their own goals and was likely influenced by factors such as health literacy and previous health care experiences. Also, there might be within-country cultural differences related to individual factors including ethnicity and sociodemographics affecting goals. It was outside the scope of this study to examine that perspective. There was little time to practice the skills of goal setting during the GLA:D Back clinician training, where the concept of goal setting and the practical process was taught all in a 30-minutes session. For clinicians with no or little experience with goal setting that is most likely not sufficient, and some clinicians have indeed expressed challenges with the goal setting dialogue [39, 40]. It would be helpful for future research to explore more comprehensive training and other didactic methods, and what it takes for clinicians to gain confidence in their competences working with goal setting.

Another potential limitation of the study was that only one person coded the Canadian data according to ICF components and SMART adherence. However, from coding of the Danish data it appeared that agreement between coders was very high and therefor risk of errors was considered low.

Goal setting is promoted as an important element of patient-centred care and behaviour change amongst patients with chronic conditions [8, 41, 42]. There is some evidence from musculoskeletal care that health coaching including patient-led goal setting may positively affect engagement in physical activity and patient outcomes [13–15]. For goals to be a helpful part of supporting self-management they should reflect patient values and promote a focus on modifiable aspects of what may facilitate and hinder goal achievement [39, 43, 44]. Pain goals can potentially hinder goal achievement if these make patients wait for reduced pain before other good things can happen [43]. Still, it may be questioned if the promotion of specific and time-bound goals is optimal even when focusing on activity and participation. The SMART approach makes it very clear what to aim for, the downside of which is that it is easy to fail and thus loose motivation [45]. Also, focusing on what people want to achieve in terms of activity may take away focus on why a change is wanted. Thus, it should be explored if alternatives to SMART may better facilitate value-based goal setting focusing on intrinsic motivation for behavior change [45]. One approach might be ‘Motivational Interviewing’, which is a person-centered, goal-directive counselling method [46]. It emphasizes focus on values and eliciting motivation to a larger degree than what SMART in itself provides but comes with higher demands for clinician communication training. Also, the value of goal setting as a tool for sustained self-management might be increased by teaching patients the skills to define and adjust goals themselves. This is not part of GLA:D Back.

The available evidence to inform how goal setting is optimally integrated in clinical practice and to determine effects of goal setting is sparse. Use of goal setting to support self-management of LBP needs thorough investigation of systematically developed goal setting interventions. Our results show that patients with long-lasting LBP pursue many different goals not necessarily captured in commonly used patient specific measurement tools and to some extent varying between sexes and age groups. Such knowledge can inform the development of self-management support tools and interventions to ensure that diverse needs are met.

### Conclusion

Goal setting was feasible in routine primary care of people with LBP attending a structured education and exercise program. Participants reported different types of goals that were mostly related to activities. Danish and Canadian patients identified similar goals with few differences across sex and age groups. The diversity in goals stresses the importance of offering patient-centred care that focusses on what is important to individual patients.

## Data Availability

Data is available from the corresponding author upon reasonable request.

## Abbreviations

GLA:D: Good Life with OsteoArthritis in Denmark. The program was originally developed for knee and hip OA. GLA:D Back only uses the abbreviation
ICF: The International Classification of Functioning, Disability and Health
LBP: Low Back Pain
SMART: Specific, Measurable, Achievable, Relevant, and Time-bound
